# Supplementary material for: Circulating Short-Chain Fatty Acids Are Positively Associated with Adiposity Measures in Chinese Adults
Source: Nutrients. 2020 Jul 17;12(7):2127. doi: 10.3390/nu12072127 (PMC7400849; doi:10.3390/nu12072127)
Supplement: Supplementary file 1 [file nutrients-12-02127-s001.pdf]

Online Supplementary Material

Circulating Short-chain Fatty Acids are Positively Associated with Adiposity Measures in Chinese Adults

Yiqing Wang, Huijun Wang, Annie Green Howard, Katie A. Meyer, Matthew C. B. Tsilimigras, Christy L. Avery, Wei Sha, Shan Sun, Jiguo Zhang, Chang Su, Zhihong Wang, Bing Zhang, Anthony A. Fodor, Penny Gordon-Larsen

Figure S1. Sample flow chart

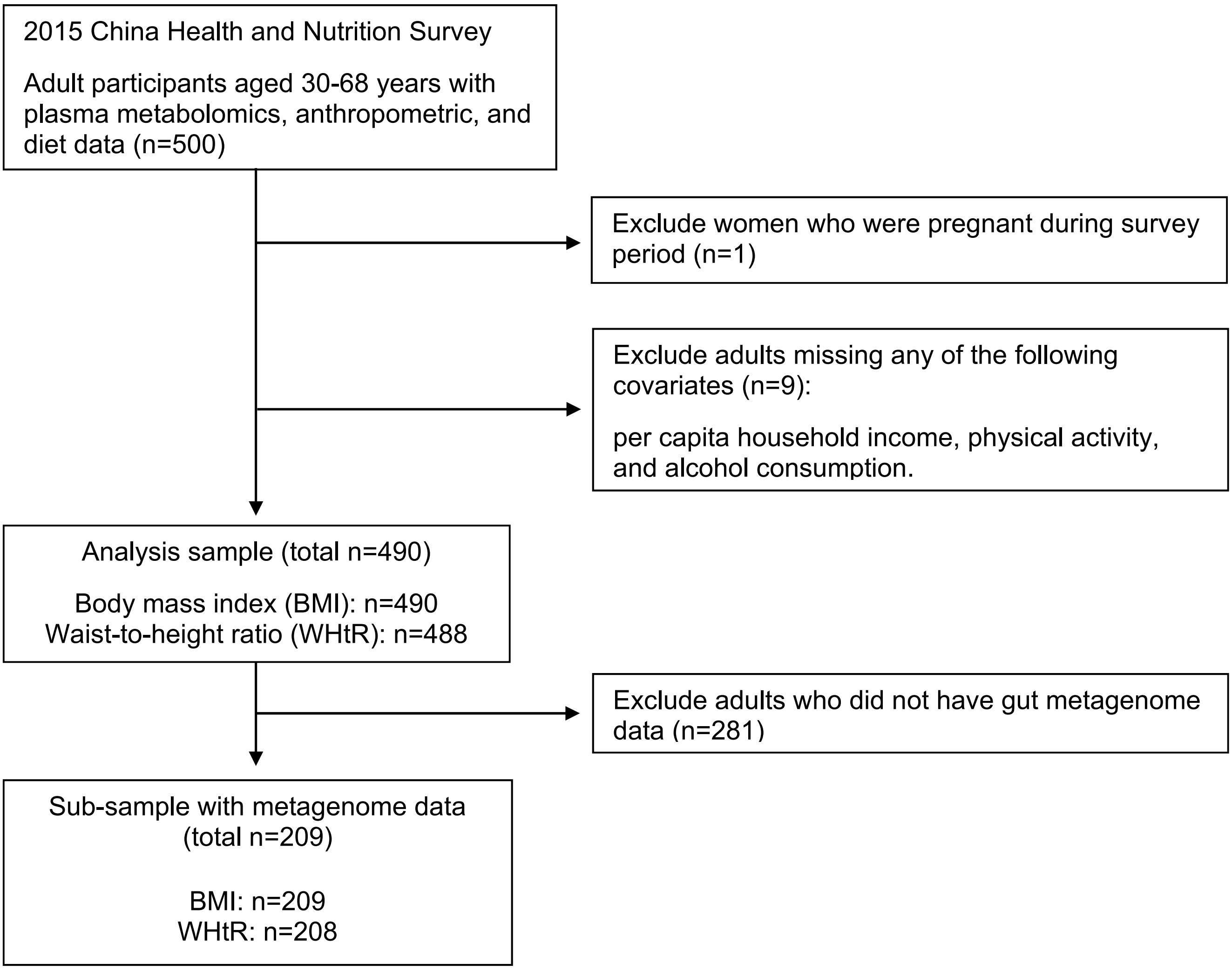

Figure S2. Sensitivity analysis of the associations between dietary precursors of short-chain fatty acids (SCFAs) and plasma SCFAs by overweight

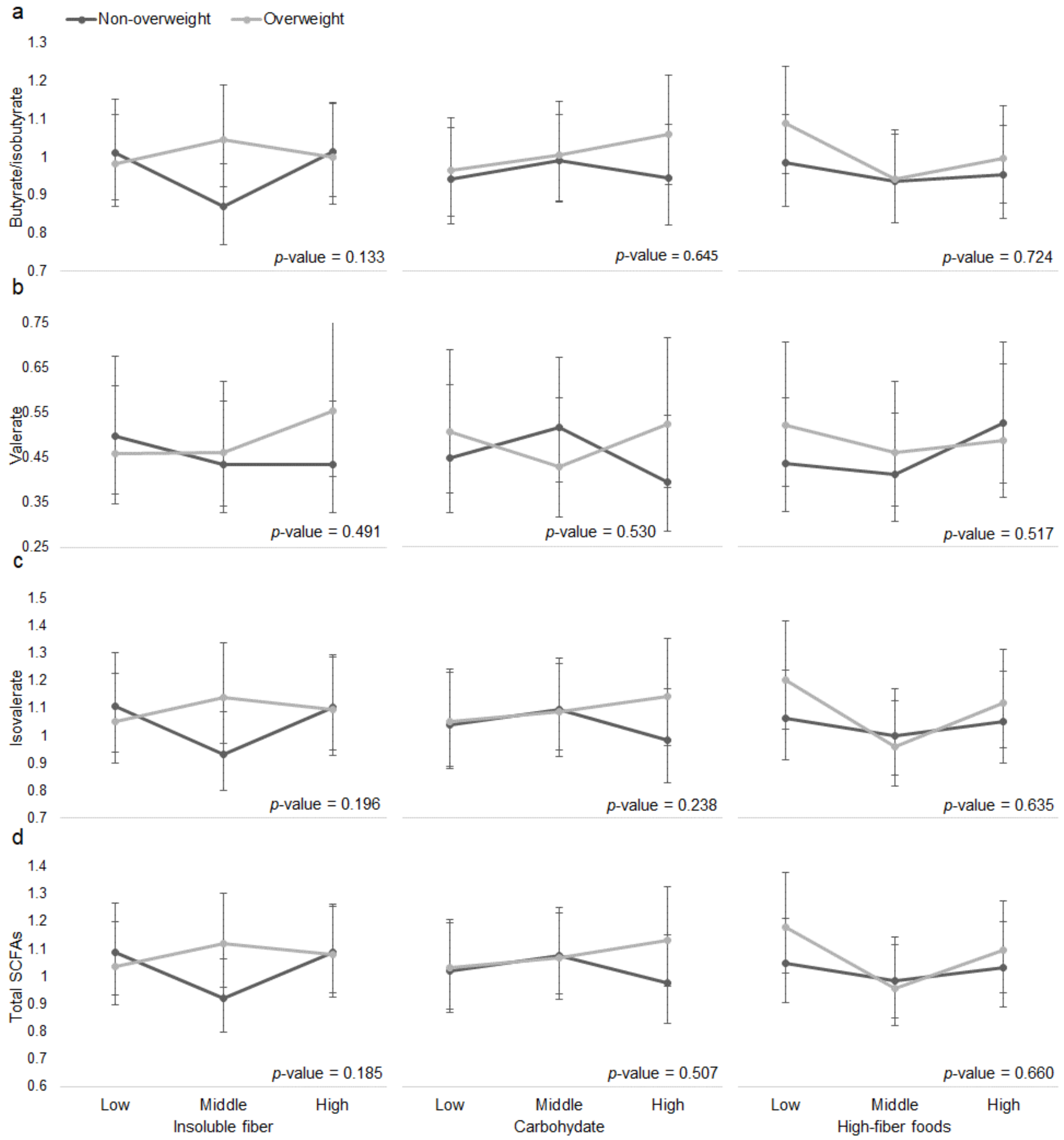

Overweight: BMI ≥24 kg/m^2. Vertical axes represent model predicted (marginal means) SCFAs abundance. Dietary intakes of insoluble fiber, total carbohydrates, and high-fiber foods were categorized by tertiles to represent low, middle, and high intakes. Linear model was adjusted for age, sex, batch run, province, urbanization, income, education, physical activity, total energy intake, alcohol, and ever smoking. For analysis of total carbohydrates and high-fiber foods, insoluble fiber intake was additionally adjusted in model. P-value for the interaction between each dietary precursor of SCFAs and overweight was derived using a Wald test. P-value>0.05 for all comparisons of plasma SCFA abundance at a given level of a dietary precursor by overweight.

Figure S3. Sensitivity analysis of the associations between dietary precursors of short-chain fatty acids (SCFAs) with plasma SCFAs by abdominal obesity

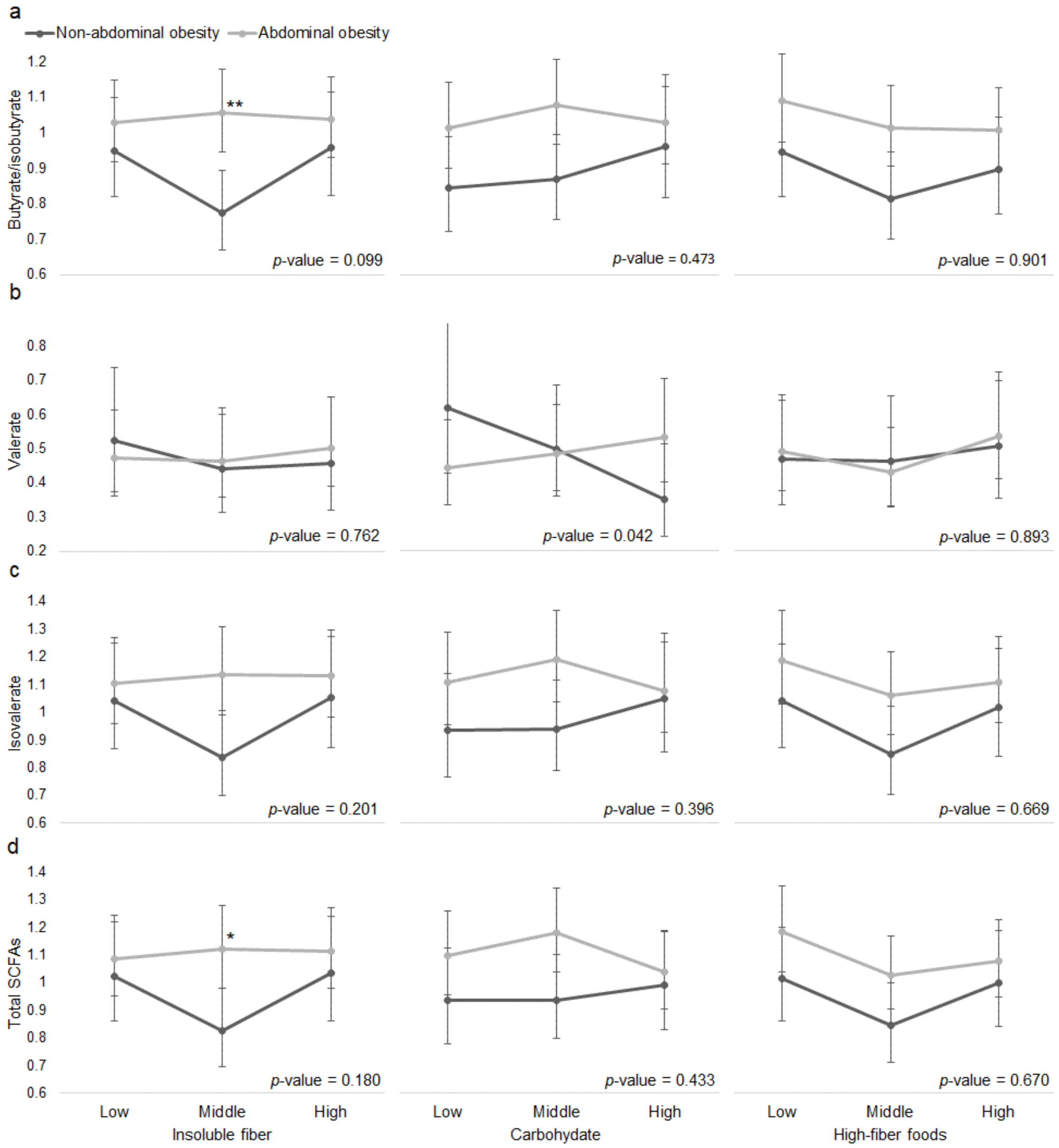

Abdominal obesity: waist-to-height ratio≥0.5. Vertical axes represent model predicted (marginal means) SCFAs abundance. Dietary intakes of insoluble fiber, total and carbohydrate, and high-fiber foods were categorized by tertiles to represent low, middle, and high intakes. Linear model was adjusted for age, sex, batch run, province, urbanization, income, education, physical activity, total energy intake, alcohol, and ever smoking. For analysis of total carbohydrate and high-fiber foods, insoluble fiber intake was additionally adjusted in model. P-value for the interaction between each dietary precursor of SCFAs and AOB was derived using a Wald test. *, p-value<0.5; **, p-value <0.01 for comparisons of plasma SCFAs abundance at a given level of dietary precursor by abdominal obesity.

Table S1. Foods included in the high-fiber food group

Food types	Sample foods
Whole grains	Buckwheat flour, foxtail millet, pea starch noodle, tartarian buckwheat flour, yellow corn (grain, grits, flour)
Legumes	Broad bean, soybean curd (soft, semisoft, slab), red bean
Starchy roots	Lotus root, sweet potato, taro, whit potato, winged yam, yam, yam bean
Vegetables	Amaranth, asparagus, bamboo shoot, bitter melon, bok choy, broccoli, cabbage, carrot, cauliflower, celery, chives, corn (fresh), cucumber, eggplant, garlic stalk, hispid yam leaf, hyacinth bean (green), hot pepper, kidney bean (green), lettuce, lily, mung bean sprounts, mustard root, pumpkin, pumpkin sprouts, radish, radish leaf, snap pea, soybean sprouts, spinach, sweet pepper, tomato, turnip, water spinach, winter melon, yardlong cowpea
Mushrooms/seaweed	Button mushroom, “gold needle” mushroom, kelp, laver, oyster mushroom, shitake mushroom, silver ear fungus, wood ear fungus
Fruits	Apple, banana, casaba, date, dragon fruit, durian, gooseberry, grape, jujube, kiwi fruit, longan, orange, pear, persimmon, pitaya, pomegranate, pomelo, tangerine, watermelon
Nuts/seeds	Chestnut, lotus seed, peanut, pumpkin seed, sesame (black, white), sunflower seed, walnut, watermelon seed

Table S2. Gut microbiota that are found to have abilities to produce short-chain fatty acids (SCFAs) in previous studies and identified in the metagenome analysis sample

Phylum	Family	Species	Acetate[1]	Butyrate[1]	Propionate[1]	References
		**Present in at least 25% of the sample**				
Actinobacteria	Bifidobacteriaceae	*Bifidobacterium longum*	Y	Y	Y	[1,2]
Bacteroidetes	Bacteroidaceae	*Bacteroides uniformis*			Y	[3]
		Bacteroides vulgatus			Y	[3,4]
	Prevotellaceae	*Prevotella copri*			Y	[3]
	Rikenellaceae	*Alistipes putredinis*		Y	Y	[3,5]
Firmicutes	Erysipelotrichaceae	*Clostridium innocuum*		Y		[6]
		Eubacterium biforme		Y		[3,5,6]
	Eubacteriaceae	*Eubacterium hallii*		Y	Y	[3-10]
		Eubacterium ramulus		Y		[6]
		Eubacterium rectale		Y		[1,3-5,7,9]
		Eubacterium ventriosum		Y		[1,6]
	Lachnospiraceae	*Anaerostipes hadrus*		Y		[3,4]
		Coprococcus catus		Y	Y	[3-6]
		Coprococcus comes		Y		[4,5]
		Lachnospiraceae bacterium 5_1_63FAA		Y		[5]
		Roseburia hominis	Y	Y		[11]
		Roseburia intestinalis		Y	Y	[3-6,9,12]
		Roseburia inulinivorans		Y		[3-5,7]

		Ruminococcus gnavus			Y	[4,13]
		Ruminococcus obeum			Y	[4]
		Ruminococcus torques			Y	[4]
	Lactobacillaceae	*Lactobacillus gasseri*			Y	[2]
	Ruminococcaceae	*Faecalibacterium prausnitzii*		Y		[1,3,4,6,7,9,12,14]
		Ruminococcus bromii	Y			[15]
	Veillonellaceae	*Dialister invisus*			Y	[3]
		Megamonas funiformis	Y		Y	[16]
		Veillonella parvula			Y	[4]
		**Present in less than 25% of the sample**				
Actinobacteria	Bifidobacteriaceae	*Bifidobacterium adolescentis*	Y			[10]
		Bifidobacterium bifidum	Y		Y	[2]
Bacteroidetes	Bacteroidaceae	*Bacteroides thetaiotaomicron*	Y		Y	[4,14]
	Porphyromonoadaceae	*Odoribacter splanchnicus*		Y		[5]
		Porphyromonas gingivalis		Y		[5]
Firmicutes	Clostridiaceae	*Clostridium beijerinckii*		Y		[6]
		Clostridium butyricum		Y		[6]
		Clostridium sp. L2-50		Y		[4]
		Clostridium symbiosum		Y		[5,6]
	Eubacteriaceae	*Anaerofustis stercorihominis*		Y		[5]
		Eubacterium limosum	Y	Y		[6,17,18]

	Erysipelotrichaceae	*Eubacterium cylindroides*		Y		[6]
		Eubacterium dolichum		Y		[6]
	Lachnospiraceae	*Anaerostipes caccae*		Y		[5-7,9,10,19]
		Blautia hydrogenotrophica	Y			[15]
		Butyrivibrio crossotus		Y		[5,6]
		Coprococcus eutactus		Y		[4-6]
	Lactobacillaceae	*Lactobacillus acidophilus*	Y	Y	Y	[20]
		Lactobacillus rhamnosus			Y	[2]
	Peptostreptococcaceae	*Clostridium difficile*		Y		[5,6]
	Ruminococcaceae	*Anaerotruncus colihominis*		Y		[5]
		Ruminococcaceae bacterium D16		Y		[5]
		Subdoligranulum variabile		Y		[3,5]
	Veillonellacea	*Megasphaera elsdenii*			Y	[4]
Fusobacteria	Fusobacteriaceae	*Fusobacterium mortiferum*		Y		[5]
		Fusobacterium nucleatum		Y		[5]
		Fusobacterium ulcerans		Y		[5]
		Fusobacterium varium		Y		[5]
Verrucomicrobia	Verrucomicrobiaceae	*Akkermansia muciniphila*	Y		Y	[3,21]

[1] “Y” indicates that the microbiota produces the given SCFA and empty cell indicates that there is no evidence showing that the microbiota produces the given SCFA.

Table S3. The interaction between dietary precursors of short-chain fatty acids (SCFAs) and overweight in models of SCFAs [% change (95% CI)], corresponding to Figure 1[1]

n=490	Butyrate/isobutyrate	Valerate	Isovalerate	Total SCFAs
		**Insoluble fiber (ref. = low)[2]**		
Middle	-15.45 (-28.4, -0.15)*	-4.23 (-35.24, 41.63)	-18.02 (-33.48, 1.05)	-17.64 (-32.17, 0.7)
High	1.47 (-14.54, 20.47)	0.87 (-32.66, 51.09)	1.47 (-18.24, 25.92)	1.4 (-17.07, 24.83)
Middle X Overweight	25.71 (0.29, 57.55)*	7.83 (-36.62, 83.46)	31.45 (-1.05, 74.62)	30.13 (-0.69, 70.53)
High X Overweight	2.87 (-17.84, 28.79)	19.41 (-29.64, 102.64)	6.38 (-19.81, 41.12)	5.7 (-18.77, 38.51)
Interaction p-value[3]	0.095	0.803	0.140	0.131
		**Carbohydrate (ref. = low)[2]**		
Middle	4.89 (-11.35, 24.1)	22.43 (-17.39, 81.44)	4.17 (-15.66, 28.66)	4.25 (-14.74, 27.46)
High	-4.37 (-20.71, 15.34)	-8.9 (-41.23, 41.21)	-10.78 (-29.48, 12.89)	-9.87 (-27.8, 13.29)
Middle X Overweight	0.08 (-20.31, 25.7)	-30.31 (-59.11, 18.77)	0.91 (-24.2, 34.34)	0.7 (-23.16, 31.95)
High X Overweight	12.38 (-10.4, 40.95)	12.18 (-33.96, 90.54)	19.29 (-10.24, 58.52)	18.92 (-9.25, 54.76)
Interaction p-value[3]	0.508	0.188	0.391	0.372
		**High-fiber foods (ref. = low)[2]**		
Middle	-7.4 (-21.45, 9.17)	4.64 (-28.92, 54.04)	-8.02 (-25.17, 13.06)	-7.98 (-24.21, 11.73)
High	-5.04 (-20.02, 12.74)	21.03 (-19.14, 81.13)	-4.25 (-22.79, 18.73)	-4.74 (-22.08, 17.28)
Middle X Overweight	-7.72 (-26.31, 15.55)	-10.56 (-47.27, 51.7)	-14.76 (-35.7, 13)	-12.94 (-33.57, 13.29)
High X Overweight	-1.63 (-21.47, 23.22)	-27.02 (-57.01, 23.88)	-1.46 (-25.7, 30.69)	-1.38 (-24.74, 28.34)
Interaction p-value[3]	0.761	0.497	0.472	0.527

[1] BMI ≥24 kg/m^2. The abundance of each plasma SCFAs and the sum of the all three SCFAs were $\log_2$ transformed. The % change relative to the reference level was calculated using the following formula: $(2^{(\beta)}-1)*100\%$, where β was the linear model coefficient.

Model was adjusted for age, sex, batch, province, urbanization, income, education, total energy, insoluble fiber intake, physical activity, smoking, and alcohol intake. *, % change p-value<0.05, **, % change p-value<0.01

[2] Dietary intakes were measured by 3-consecutive 24h dietary recalls and household food inventories. High-fiber foods was calculated as the sum of whole grains, legumes, starchy roots, vegetables, mushrooms/seaweeds, fruits, nuts/seeds. Insoluble fiber, carbohydrate, and high-fiber food score were categorized by tertiles to represent low, middle, and high intakes.

[3] The statistical significance of the interaction term between dietary factors and overweight was estimated using a Wald test that compared models with and without the interaction term.

Table S4. The interaction between dietary precursors of short-chain fatty acids (SCFAs) and abdominal obesity in models of SCFAs [% change (95% CI)], corresponding to Figure 2[1]

n=488	Butyrate/isobutyrate	Valerate	Isovalerate	Total SCFAs
		**Insoluble fiber (ref. = low)[2]**		
Middle	-18.42 (-33.00, -0.66)*	-9.96 (-43.57, 43.66)	-19.07 (-36.9, 3.8)	-18.77 (-35.83, 2.81)
High	6.31 (-12.88, 29.73)	1.72 (-36.57, 63.13)	9.63 (-14.76, 41)	9.43 (-14.14, 38.51)
Middle X Abdominal obesity	23.88 (-2.03, 56.63)	14.72 (-34.24, 100.13)	23.52 (-8.17, 66.14)	23.11 (-6.7, 63.58)
High X Abdominal obesity	-5.15 (-24.96, 19.9)	8.45 (-37.8, 89.1)	-7.08 (-30.9, 24.95)	-6.7 (-38.87, 23.97)
Interaction p-value[3]	0.063	0.888	0.150	0.132
		**Carbohydrate (ref. = low)[2]**		
Middle	3.24 (-15.5, 26.14)	-17.26 (-48.32, 32.47)	-0.02 (-22.34, 28.71)	0.14 (-21.00, 27.46)
High	9.64 (-11.83, 36.35)	-41.01 (-64.65, -1.56)*	5.98 (-19.49, 39.51)	5.7 (-18.21, 37.55)
Middle X Abdominal obesity	2.91 (-18.84, 30.49)	36.07 (-22.1, 137.67)	7.41 (-20.38, 44.9)	7.18 (-19.34, 42.41)
High X Abdominal obesity	-11.21 (-30.08, 12.75)	100.19 (14.22, 250.87)*	-12.13 (-34.99, 18.75)	-11.12 (-33.1, 18.92)
Interaction p-value[3]	0.428	0.052	0.404	0.428
		**High-fiber foods (ref. = low)[2]**		
Middle	-14.88 (-29.94, 3.41)	10.78 (-30.14, 75.66)	-17.55 (-35.48, 5.37)	-17.07 (-34.02, 4.97)
High	-3.71 (-21.12, 17.54)	8.02 (-32.65, 73.24)	-0.95 (-22.97, 27.34)	-1.38 (-22.62, 24.83)
Middle X Abdominal obesity	6.66 (-15.49, 34.62)	-14.69 (-50.85, 48.09)	4.3 (-22.22, 39.86)	4.25 (-21, 37.55)
High X Abdominal obesity	-4.84 (-24.57, 20.03)	-2.69 (-43.87, 68.69)	-7.95 (-31.31, 23.35)	-7.34 (-29.78, 22.26)
Interaction p-value[3]	0.634	0.835	0.702	0.695

[1] Waist-to-height ratio ≥0.5. The abundance of each SCFAs and the sum of the all three SCFAs were $\log_2$ transformed. The % change relative to the reference level was calculated using the following formula: (2^(β)-1)*100%, where β was the linear model

coefficient. Model was adjusted for age, sex, batch, province, urbanization, income, education, total energy, insoluble fiber, physical activity, smoking, and alcohol intake. *, % change p-value<0.05, **, % change p-value<0.01.

[2] Dietary intakes were measured by 3-consecutive 24h dietary recalls and household food inventories. High-fiber foods was calculated as the sum of whole grains, legumes, starchy roots, vegetables, mushrooms/seaweeds, fruits, nuts/seeds. Insoluble fiber, carbohydrate, and high-fiber food score were categorized by tertiles to represent low, middle, and high intakes.

[3] The statistical significance of the interaction term between dietary factors and abdominal obesity was estimated using a Wald test that compared models with and without the interaction term.

Table S5. The associations between individual foods and short-chain fatty acids (SCFAs) by overweight (n=490)[1]

	Butyrate/isobutyrate			Valerate			Isovalerate			Total SCFAs		
	Inter-action p-value[2]	Inter-action q-value[2]	% change (95% CI)	Inter-action p-value[2]	Inter-action q-value[2]	% change (95% CI)	Inter-action p-value[2]	Inter-action q-value[2]	% change (95% CI)	Inter-action p-value[2]	Inter-action q-value[2]	% change (95% CI)
**Wholegrains (ref. = non-consumer)[3]**												
Non-overweight	0.64	0.96	-9.24 (-30.92, 19.26)	0.36	0.92	25.87 (-33.52, 138.32)	0.79	0.96	-11.06 (-36.91, 25.39)	0.67	0.96	-10.73 (-35.56, 23.66)
Overweight			-17.71 (-39.27, 11.51)			-18.75 (-60.07, 65.35)			-16.92 (-43.31, 21.76)			-19.65 (-44.05, 15.4)
**Legumes (ref. = below median)[3]**												
Non-overweight	0.56	0.96	7.66 (-5.8, 23.04)	0.06	0.56	21.84 (-10.74, 66.31)	0.42	0.92	9.61 (-7.34, 29.65)	0.51	0.95	7.33 (-8.51, 25.93)
Overweight			1.82 (-11.17, 16.73)			-19.54 (-41.47, 10.59)			-0.45 (-16.15, 18.2)			-0.35 (-15.41, 17.39)
**Starchy roots (ref. = below median)[3]**												
Non-overweight	0.89	0.96	-5.47 (-17.1, 7.79)	0.8	0.96	-5.75 (-30.65, 28.11)	0.74	0.96	-3.33 (-18.05, 14.02)	0.7	0.96	-4.06 (-18.02, 12.28)
Overweight			-6.67 (-18.45, 6.81)			-10.88 (-34.98, 22.17)			-7 (-21.52, 10.21)			-8.1 (-21.77, 7.96)
**Vegetables (ref. = below median)[3]**												
Non-overweight	0.44	0.92	-3.44 (-15.31, 10.1)	0.51	0.95	7.73 (-20.7, 46.35)	0.32	0.92	-4.62 (-19.12, 12.47)	0.34	0.92	-4.58 (-18.58, 11.82)

Overweight			3.95 (-9.21, 19)			-6.94 (-32.15, 27.65)			7.46 (-9.34, 27.38)			6.34 (-9.66, 25.19)
**Mushrooms/seaweeds (ref. = non-consumer)[3]**												
Non-overweight	0.32	0.92	3.86 (-9.6, 19.31)	0.32	0.92	-16.28 (-39.44, 15.74)	0.47	0.93	7.97 (-9.31, 28.54)	0.4	0.92	5.53 (-10.63, 24.61)
Overweight			-6.01 (-18.84, 8.86)			5.94 (-24.8, 49.26)			-1.53 (-18.13, 18.43)			-4.55 (-20.02, 13.91)
**Fruits (ref. = non-consumer)[3]**												
Non-overweight	0.89	0.96	-6.54 (-18.43, 7.08)	0.04	0.45	4.8 (-23.61, 43.79)	0.9	0.96	-5.36 (-20.25, 12.31)	0.9	0.96	-7.12 (-21.21, 9.49)
Overweight			-7.76 (-19.58, 5.79)			-32.81 (-51.15, -7.58)*			-6.76 (-21.54, 10.8)			-8.47 (-22.41, 7.98)
**Nuts/seeds (ref. = non-consumer)[3]**												
Non-overweight	0.69	0.96	11.37 (-5.64, 31.45)	0.78	0.96	-1.14 (-32.92, 45.71)	0.61	0.96	17.98 (-4.19, 45.27)	0.64	0.96	16.7 (-4.22, 42.2)
Overweight			6.19 (-10.59, 26.11)			6.78 (-28.6, 59.67)			9.33 (-11.9, 35.69)			9.19 (-11.07, 34.06)

[1] BMI ≥24 kg/m^2. The abundance of each SCFAs and the sum of the all three SCFAs were $\log_2$ transformed. The % change relative to the reference level was calculated using the following formula: (2^(β)-1)*100%, where β was the linear model coefficient. Model was adjusted for age, sex, batch, province, urbanization, income, education, total energy, insoluble fiber, physical activity, smoking, and alcohol intake. *, % change p-value<0.05.

[2] The statistical significance of the interaction term between dietary factors and overweight was estimated using a Wald test that compared models with and without the interaction term. Q-value was false-discovery rate adjusted p-value.

[3] Dietary intakes were measured by 3-consecutive 24h dietary recalls and household food inventories. Foods consumed by more than and less than 50% of the analysis sample were categorized by median and yes/no consumers, respectively.

Table S6. The associations between individual foods and short-chain fatty acids (SCFAs) by abdominal obesity (n=488)[1]

	Butyrate/isobutyrate			Valerate			Isovalerate			Total SCFAs		
	Inter-action p-value[2]	Inter-action q-value[2]	% change (95% CI)	Inter-action p-value[2]	Inter-action q-value[2]	% change (95% CI)	Inter-action p-value[2]	Inter-action q-value[2]	% change (95% CI)	Inter-action p-value[2]	Inter-action q-value[2]	% change (95% CI)
**Wholegrains (ref. = non-consumer)[3]**												
Non-abdominal obesity	0.10	0.63	9.32 (-22.51, 54.23)	0.01	0.13	164.02 (17.88, 491.34)*	0.16	0.88	10.65 (-28.38, 70.95)	0.10	0.63	11.82 (-25.89, 68.71)
Abdominal obesity			-23.35 (-40.14, -1.85)*			-36.26 (-64.28, 13.74)			-24.58 (-44.82, 3.07)			-26.45 (-45.27, -1.17)*
**Legumes (ref. = below median)[3]**												
Non-abdominal obesity	0.45	0.92	9.81 (-6.22, 28.57)	0.98	0.98	-0.07 (-31.07, 44.89)	0.42	0.92	11.39 (-8.72, 35.94)	0.53	0.96	8.33 (-10.35, 30.9)
Abdominal obesity			1.97 (-9.38, 14.73)			-0.53 (-24.65, 31.33)			0.83 (-13.13, 17.02)			0.73 (-12.61, 16.12)
**Starchy roots (ref. = below median)[3]**												
Non-abdominal obesity	0.95	0.97	-5.83 (-19.74, 10.48)	0.42	0.92	5.93 (-27.27, 54.28)	0.87	0.96	-3.99 (-21.54, 17.48)	0.82	0.96	-4.49 (-21.16, 15.71)
Abdominal obesity			-6.44 (-16.56, 4.91)			-12.1 (-32.86, 15.09)			-5.98 (-18.64, 8.66)			-7.07 (-18.98, 6.59)
**Vegetables (ref. = below median)[3]**												
Non-abdominal obesity	0.95	0.97	-0.16 (-14.69, 16.84)	0.83	0.96	-4.23 (-33.82, 38.6)	0.82	0.96	3.13 (-15.43, 25.76)	0.89	0.96	1.99 (-15.57, 23.2)
Abdominal obesity			0.44 (-10.51, 12.74)			0.67 (-23.26, 32.06)			0.33 (-13.27, 16.07)			0.35 (-12.88, 15.6)
**Mushrooms/seaweeds (ref. = non-consumer)[3]**												

Non-abdominal obesity	0.36	0.92	5.21 (-11.27, 24.76)	0.88	0.96	-8.7 (-38.85, 36.31)	0.34	0.92	12.2 (-9.5, 39.1)	0.20	0.92	11.7 (-8.99, 37.08)
Abdominal obesity			-4.52 (-15.72, 8.17)			-5.13 (-29.26, 27.23)			-1.31 (-15.68, 15.52)			-5 (-18.3, 10.47)
**Fruits (ref. = non-consumer)[3]**												
Non-abdominal obesity	0.28	0.92	-0.48 (-15.42, 17.11)	0.00	0.07	39.98 (-4.17, 104.47)	0.36	0.92	1.05 (-17.74, 24.13)	0.33	0.92	-0.78 (-18.4, 20.66)
Abdominal obesity			-10.49 (-20.55, 0.84)			-33.35 (-49.51, -12.03)**			-9.66 (-22.3, 5.04)			-11.35 (-23.34, 2.52)
**Nuts/seeds (ref. = non-consumer)[3]**												
Non-abdominal obesity	0.09	0.63	24.46 (1.69, 52.34)	0.91	0.96	6.65 (-33.84, 71.91)	0.02	0.38	43.57 (11.35, 85.13)	0.03	0.44	38.63 (8.84, 76.58)**
Abdominal obesity			0.39 (-13.32, 16.26)			2.98 (-27.2, 45.65)			-0.61 (-17.36, 19.54)			0.37 (-15.75, 19.58)

[1] Waist-to-height ratio ≥0.5. The abundance of each SCFAs and the sum of the all three SCFAs were $\log_2$ transformed. The % change relative to the reference level was calculated using the following formula: (2^(β)-1)*100%, where β was the linear model coefficient. Model was adjusted for age, sex, batch, province, urbanization, income, education, total energy, insoluble fiber, physical activity, smoking, and alcohol intake. *, % change p-value<0.05; **, % change p-value<0.01.

[2] The statistical significance of the interaction term between dietary factors and abdominal obesity was estimated using a Wald test that compared models with and without the interaction term. Q-value was false-discovery rate adjusted p-value.

[3] Dietary intakes were measured by 3-consecutive 24h dietary recalls and household food inventories. Foods consumed by more than and less than 50% of the analysis sample were categorized by median and yes/no consumers, respectively.

Table S7. The associations between each 56 microbial short-chain fatty acid (SCFA) producers with body mass index (BMI) and waist-to-height ratio (WHtR)[1]

Species	BMI (n=209)			WHtR (n=208)			Rare[3]
	β (95% confidence interval)	p-value	q-value[2]	β (95% confidence interval)	p-value	q-value[2]	
Bifidobacterium longum	-0.03 (-0.24, 0.18)	0.78	1.00	3.E-06 (-4.E-03, 4.E-03)	1.00	1.00	N
Bacteroides thetaiotaomicron	-0.32 (-0.66, 0.01)	0.06	0.48	-7.E-03 (-0.01, -1.E-03)	0.02	0.24	N
Bacteroides uniformis	-0.07 (-0.4, 0.25)	0.66	1.00	-3.E-03 (-0.01, 3.E-03)	0.38	0.84	N
Bacteroides vulgatus	0.05 (-0.33, 0.42)	0.80	1.00	-3.E-04 (-0.01, 0.01)	0.92	1.00	N
Prevotella copri	0.08 (-0.16, 0.32)	0.51	0.88	3.E-03 (-2.E-03, 0.01)	0.25	0.84	N
Alistipes putredinis	0.15 (-0.14, 0.43)	0.31	0.84	3.E-04 (-5.E-03, 0.01)	0.92	1.00	N
Lactobacillus gasseri	-0.07 (-0.44, 0.31)	0.73	1.00	-1.E-04 (-0.01, 0.01)	0.97	1.00	N
Eubacterium hallii	0.54 (0.22, 0.87)	1.E-03	0.07	9.E-03 (3.E-03, 0.01)	0.01	0.09	N
Eubacterium ramulus	0.21 (-0.09, 0.51)	0.17	0.84	3.E-03 (-3.E-03, 0.01)	0.30	0.84	N
Eubacterium rectale	0.31 (0.08, 0.54)	0.01	0.14	6.E-03 (2.E-03, 0.01)	0.01	0.09	N
Eubacterium ventriosum	0.01 (-0.29, 0.31)	0.95	1.00	1.E-04 (-0.01, 0.01)	0.97	1.00	N
Anaerostipes hadrus	0.22 (-0.17, 0.61)	0.27	0.84	1.E-03 (-0.01, 0.01)	0.78	1.00	N
Ruminococcus gnavus	-0.04 (-0.29, 0.22)	0.78	1.00	3.E-05 (-5.E-03, 5.E-03)	0.99	1.00	N
Ruminococcus obeum	0.53 (-0.04, 1.11)	0.07	0.52	6.E-03 (-5.E-03, 0.02)	0.28	0.84	N
Ruminococcus torques	0.61 (-0.09, 1.31)	0.09	0.62	9.E-03 (-4.E-03, 0.02)	0.18	0.84	N
Coprococcus catus	0.03 (-0.25, 0.31)	0.83	1.00	2.E-03 (-3.E-03, 0.01)	0.44	0.84	N
Coprococcus comes	0.01 (-0.24, 0.27)	0.92	1.00	-2.E-05 (-5.E-03, 5.E-03)	0.99	1.00	N
Lachnospiraceae bacterium 5 1 63FAA	0.2 (-0.18, 0.58)	0.29	0.84	7.E-04 (-0.01, 0.01)	0.85	1.00	N
Roseburia hominis	0.17 (-0.13, 0.46)	0.27	0.84	2.E-04 (-0.01, 0.01)	0.95	1.00	N
Roseburia intestinalis	0.23 (-0.06, 0.51)	0.12	0.73	3.E-04 (-5.E-03, 0.01)	0.90	1.00	N
Roseburia inulinivorans	0.11 (-0.19, 0.41)	0.46	0.84	1.E-03 (-5.E-03, 0.01)	0.72	1.00	N
Faecalibacterium prausnitzii	0.17 (-0.23, 0.57)	0.40	0.84	4.E-03 (-4.E-03, 0.01)	0.34	0.84	N

Ruminococcus bromii	-0.13 (-0.34, 0.08)	0.23	0.84	-2.E-03 (-0.01, 2.E-03)	0.39	0.84	N
Clostridium innocuum	-0.05 (-0.57, 0.47)	0.85	1.00	2.E-04 (-0.01, 0.01)	0.97	1.00	N
Eubacterium biforme	0.08 (-0.14, 0.31)	0.47	0.86	3.E-03 (-1.E-03, 0.01)	0.15	0.80	N
Dialister invisus	-0.16 (-0.48, 0.17)	0.34	0.84	-3.E-03 (-0.01, 3.E-03)	0.37	0.84	N
Megamonas funiformis	0.45 (0.14, 0.76)	0.01	0.09	6.E-03 (-8.40E-05, 0.01)	0.05	0.46	N
Veillonella parvula	-0.16 (-0.55, 0.23)	0.42	0.84	-2.E-03 (-0.01, 5.E-03)	0.53	0.88	N
Akkermansia muciniphila	0.06 (-0.19, 0.31)	0.66	1.00	1.E-03 (-4.E-03, 0.01)	0.69	1.00	N
Bifidobacterium adolescentis	-0.09 (-1.33, 1.15)	0.88	1.00	7.E-03 (-0.02, 0.03)	0.56	0.91	Y
Bifidobacterium bifidum	-0.89 (-2.46, 0.68)	0.26	0.84	-1.E-02 (-0.04, 0.02)	0.45	0.84	Y
Odoribacter splanchnicus	-0.26 (-1.49, 0.98)	0.68	1.00	3.E-03 (-0.02, 0.03)	0.83	1.00	Y
Porphyromonas gingivalis	-1.5 (-4.44, 1.45)	0.32	0.84	2.E-02 (-0.03, 0.08)	0.43	0.84	Y
Lactobacillus acidophilus	0.46 (-4.14, 5.06)	0.84	1.00	2.E-02 (-0.07, 0.1)	0.68	1.00	Y
Lactobacillus rhamnosus	0.01 (-1.37, 1.4)	0.98	1.00	-8.E-03 (-0.03, 0.02)	0.54	0.88	Y
Clostridium beijerinckii	0.22 (-6.5, 6.94)	0.95	1.00	-7.E-02 (-0.19, 0.06)	0.29	0.84	Y
Clostridium butyricum	-1.14 (-3.86, 1.57)	0.41	0.84	-4.E-02 (-0.09, 0.01)	0.15	0.80	Y
Clostridium sp. L2 50	0.39 (-1.85, 2.62)	0.73	1.00	2.E-02 (-0.02, 0.06)	0.37	0.84	Y
Clostridium symbiosum	-2.08 (-3.22, -0.94)	4.E-04	0.04	-2.E-02 (-0.05, -4.E-03)	0.02	0.27	Y
Anaerofustis stercorihominis	0.9 (-0.67, 2.48)	0.26	0.84	1.E-02 (-0.02, 0.04)	0.41	0.84	Y
Eubacterium limosum	-0.01 (-1.25, 1.23)	0.99	1.00	-9.E-03 (-0.03, 0.01)	0.41	0.84	Y
Anaerostipes caccae	-0.94 (-3.02, 1.15)	0.38	0.84	-1.E-02 (-0.05, 0.03)	0.54	0.88	Y
Blautia hydrogenotrophica	-1.61 (-3.62, 0.4)	0.12	0.73	-4.E-02 (-0.07, -2.E-04)	0.05	0.45	Y
Butyrivibrio crossotus	-0.94 (-2.82, 0.95)	0.33	0.84	-7.E-03 (-0.04, 0.03)	0.68	1.00	Y
Coprococcus eutactus	0.5 (-0.99, 1.98)	0.51	0.88	1.E-02 (-0.02, 0.04)	0.44	0.84	Y
Clostridium difficile	0.07 (-1.62, 1.77)	0.93	1.00	6.E-03 (-0.02, 0.04)	0.70	1.00	Y
Anaerotruncus colihominis	-0.93 (-2.63, 0.77)	0.28	0.84	-2.E-02 (-0.05, 0.01)	0.19	0.84	Y

Ruminococcaceae bacterium D16	-3.36 (-6.61, -0.11)	0.04	0.44	-5.E-02 (-0.11, 0.01)	0.14	0.80	Y
Subdoligranulum variabile	-0.88 (-2.69, 0.92)	0.34	0.84	-2.E-02 (-0.05, 0.02)	0.37	0.84	Y
Eubacterium cylindroides	-0.77 (-2.81, 1.28)	0.46	0.84	-4.E-03 (-0.04, 0.03)	0.83	1.00	Y
Eubacterium dolichum	0.71 (-0.65, 2.07)	0.31	0.84	3.E-03 (-0.02, 0.03)	0.79	1.00	Y
Megasphaera elsdenii	-0.41 (-4.95, 4.14)	0.86	1.00	4.E-02 (-0.04, 0.12)	0.36	0.84	Y
Fusobacterium mortiferum	-0.06 (-1.21, 1.09)	0.92	1.00	5.E-04 (-0.02, 0.02)	0.96	1.00	Y
Fusobacterium nucleatum	-0.55 (-2.1, 0.99)	0.48	0.86	2.E-02 (-0.01, 0.04)	0.27	0.84	Y
Fusobacterium ulcerans	-0.62 (-3.59, 2.35)	0.68	1.00	-9.E-03 (-0.06, 0.05)	0.75	1.00	Y
Fusobacterium varium	-4.78 (-7.92, -1.64)	3.E-03	0.09	-6.E-02 (-0.12, -4.E-03)	0.04	0.41	Y

[1] The 56 microbial SCFA producers were selected from literature and the full list with references is in Table S2. The raw counts of each species was normalized and $\log_{10}$ transformed [22]. Model was adjusted for age, sex, batch, province, urbanization, income, education, total energy, insoluble fiber, physical activity, smoking, and alcohol intake. β coefficients are interpreted as units of BMI or WHtR associated with per 1 unit increase in $\log_{10}$ of the relative abundance of microbiota.

[2] Q-value was false-discovery rate adjusted p-value.

[3] Microbiota presented in at least 25% of the sample were denoted as non-rare microbiota and kept as continuous variables in linear regression. Microbiota present in less than 25% of the sample were denoted as rare microbiota and included as binary variables (yes/no present in the sample) in linear regression.

Table S8. The associations between the overall and the total relative abundance of 56 microbial short-chain fatty acid (SCFA) producers with plasma SCFAs (n=209).

	Overall[1]		Total[2]	
	R^2	P-value	β (95% CI)	P-value
Butyrate/isobutyrate	2E-03	0.95	-0.01 (-0.39, 0.37)	0.97
Valerate	4E-03	0.58	0.54 (-0.37, 1.46)	0.24
Isovalerate	3E-03	0.79	0.00 (-0.49, 0.48)	0.99
Total SCFAs	0.01	0.03	-0.04 (-0.49, 0.41)	0.96

The 56 microbial SCFA producers were selected from literature and the full list with references is in Table S2. The abundance of each SCFAs and the sum of the all three SCFAs were $\log_2$ transformed. The raw counts of each species and the total counts of the 56 species were normalized and $\log_{10}$ transformed [22]. Model was adjusted for age, sex, batch, province, urbanization, income, education, total energy, insoluble fiber, physical activity, smoking, and alcohol intake.

[1] R^2 and p-value were calculated using permutational multivariate analysis of variance (PERMANOVA) of all 56 species.

[2] Linear regression was performed on the total relative abundance of the 56 species.

Table S9. The associations between each 56 microbial short-chain fatty acid (SCFA) producers with plasma SCFAs (n=209)[1]

	Butyrate/isovalerate		Valerate		Isovalerate		Total SCFAs		
**Species**	**β (95% confidence interval)**	**p-value**	**β (95% confidence interval)**	**p-value**	**β (95% confidence interval)**	**p-value**	**β (95% confidence interval)**	**p-value**	**Rare[2]**
Bifidobacterium longum	0 (-0.05, 0.04)	0.84	-0.04 (-0.16, 0.07)	0.46	-0.02 (-0.08, 0.04)	0.54	-0.01 (-0.07, 0.05)	0.77	N
Bacteroides thetaiotaomicron	-0.04 (-0.12, 0.04)	0.29	0.02 (-0.16, 0.21)	0.81	-0.04 (-0.14, 0.05)	0.38	-0.05 (-0.14, 0.04)	0.27	N
Bacteroides uniformis	0 (-0.07, 0.08)	0.92	-0.13 (-0.3, 0.05)	0.17	0 (-0.09, 0.1)	0.92	0 (-0.09, 0.08)	0.92	N
Bacteroides vulgatus	-0.03 (-0.12, 0.05)	0.47	-0.08 (-0.28, 0.13)	0.46	-0.04 (-0.14, 0.07)	0.52	-0.02 (-0.12, 0.08)	0.65	N
Prevotella copri	-0.03 (-0.08, 0.03)	0.31	0 (-0.13, 0.13)	0.99	-0.03 (-0.1, 0.04)	0.42	-0.02 (-0.08, 0.05)	0.60	N
Alistipes putredinis	-0.02 (-0.08, 0.04)	0.54	0.04 (-0.11, 0.2)	0.59	-0.03 (-0.11, 0.05)	0.48	-0.03 (-0.11, 0.05)	0.45	N
Lactobacillus gasseri	-0.08 (-0.16, 0.01)	0.07	-0.03 (-0.24, 0.18)	0.78	-0.09 (-0.19, 0.02)	0.12	-0.09 (-0.2, 0.01)	0.06	N
Eubacterium hallii	0 (-0.08, 0.07)	0.93	0 (-0.18, 0.18)	0.99	-0.02 (-0.12, 0.07)	0.65	-0.02 (-0.11, 0.07)	0.72	N
Eubacterium ramulus	-0.05 (-0.11, 0.02)	0.19	0.03 (-0.14, 0.2)	0.73	-0.06 (-0.15, 0.02)	0.16	-0.05 (-0.13, 0.03)	0.21	N
Eubacterium rectale	0 (-0.05, 0.05)	0.99	0.09 (-0.04, 0.22)	0.16	0 (-0.07, 0.06)	0.93	0 (-0.06, 0.07)	0.96	N
Eubacterium ventriosum	-0.05 (-0.12, 0.02)	0.18	0.01 (-0.16, 0.18)	0.91	-0.06 (-0.15, 0.03)	0.16	-0.05 (-0.13, 0.03)	0.26	N
Anaerostipes hadrus	0 (-0.09, 0.09)	0.97	0.08 (-0.14, 0.3)	0.46	-0.01 (-0.12, 0.11)	0.89	-0.01 (-0.12, 0.1)	0.87	N
Ruminococcus gnavus	0.05 (0, 0.11)	0.07	0.05 (-0.09, 0.18)	0.52	0.07 (0, 0.14)	0.05	0.07 (0, 0.13)	0.06	N
Ruminococcus obeum	-0.11 (-0.25, 0.02)	0.10	0.15 (-0.17, 0.48)	0.35	-0.16 (-0.33, 0.01)	0.06	-0.15 (-0.31, 0.01)	0.06	N
Ruminococcus torques	0.03 (-0.13, 0.19)	0.74	0.27 (-0.12, 0.65)	0.17	0.05 (-0.16, 0.25)	0.66	0.04 (-0.15, 0.23)	0.66	N
Coprococcus catus	-0.03 (-0.09, 0.04)	0.39	-0.04 (-0.2, 0.11)	0.58	-0.04 (-0.12, 0.04)	0.28	-0.04 (-0.11, 0.04)	0.35	N
Coprococcus comes	0.02 (-0.04, 0.08)	0.55	-0.01 (-0.15, 0.13)	0.93	0.02 (-0.05, 0.09)	0.60	0.02 (-0.04, 0.09)	0.48	N
Lachnospiraceae bacterium 5 1 63FAA	0.06 (-0.03, 0.15)	0.18	0.1 (-0.11, 0.31)	0.36	0.08 (-0.03, 0.19)	0.16	0.07 (-0.03, 0.18)	0.16	N
Roseburia hominis	-0.05 (-0.11, 0.02)	0.17	0.07 (-0.1, 0.23)	0.42	-0.06 (-0.15, 0.02)	0.15	-0.05 (-0.13, 0.03)	0.20	N
Roseburia intestinalis	-0.02 (-0.08, 0.05)	0.62	-0.19 (-0.35, -0.04)	0.01	-0.02 (-0.1, 0.06)	0.59	-0.02 (-0.1, 0.06)	0.60	N

Roseburia inulinivorans	0.01 (-0.06, 0.08)	0.78	-0.11 (-0.27, 0.06)	0.21	0.01 (-0.08, 0.1)	0.83	0.01 (-0.07, 0.09)	0.76	N
Faecalibacterium prausnitzii	-0.09 (-0.18, 0)	0.06	-0.06 (-0.28, 0.16)	0.61	-0.14 (-0.25, -0.02)	0.02	-0.13 (-0.24, -0.02)	0.02	N
Ruminococcus bromii	-0.03 (-0.07, 0.02)	0.30	-0.02 (-0.14, 0.1)	0.75	-0.04 (-0.1, 0.02)	0.20	-0.04 (-0.1, 0.01)	0.12	N
Clostridium innocuum	0.02 (-0.1, 0.14)	0.70	0.25 (-0.03, 0.54)	0.08	0.04 (-0.11, 0.19)	0.64	0.04 (-0.11, 0.18)	0.62	N
Eubacterium biforme	0 (-0.05, 0.06)	0.88	0.09 (-0.03, 0.22)	0.16	0 (-0.06, 0.07)	0.89	0.01 (-0.05, 0.07)	0.78	N
Dialister invisus	-0.04 (-0.12, 0.03)	0.25	0.11 (-0.07, 0.28)	0.25	-0.06 (-0.16, 0.03)	0.19	-0.07 (-0.15, 0.02)	0.14	N
Megamonas funiformis	-0.03 (-0.1, 0.04)	0.39	0.01 (-0.16, 0.19)	0.89	-0.02 (-0.11, 0.07)	0.63	-0.02 (-0.11, 0.07)	0.63	N
Veillonella parvula	0.06 (-0.03, 0.15)	0.19	-0.03 (-0.25, 0.19)	0.80	0.1 (-0.02, 0.21)	0.09	0.08 (-0.02, 0.19)	0.13	N
Akkermansia muciniphila	-0.03 (-0.09, 0.02)	0.23	0.07 (-0.06, 0.21)	0.29	-0.05 (-0.12, 0.02)	0.19	-0.05 (-0.11, 0.02)	0.18	N
Bifidobacterium adolescentis	0.11 (-0.18, 0.39)	0.46	-0.12 (-0.82, 0.57)	0.73	0.14 (-0.22, 0.51)	0.45	0.19 (-0.15, 0.52)	0.27	Y
Bifidobacterium bifidum	0.01 (-0.35, 0.37)	0.96	-0.54 (-1.4, 0.33)	0.22	0.04 (-0.41, 0.5)	0.85	0.07 (-0.36, 0.5)	0.74	Y
Odoribacter splanchnicus	-0.12 (-0.4, 0.16)	0.39	-0.05 (-0.73, 0.63)	0.89	-0.17 (-0.53, 0.19)	0.34	-0.16 (-0.49, 0.18)	0.36	Y
Porphyromonas gingivalis	0.36 (-0.32, 1.03)	0.30	0.57 (-1.07, 2.21)	0.49	0.43 (-0.43, 1.29)	0.33	0.44 (-0.36, 1.24)	0.28	Y
Lactobacillus acidophilus	0.5 (-0.56, 1.55)	0.35	2.08 (-0.46, 4.62)	0.11	0.49 (-0.85, 1.83)	0.47	0.63 (-0.61, 1.88)	0.32	Y
Lactobacillus rhamnosus	-0.17 (-0.48, 0.15)	0.29	0.34 (-0.43, 1.1)	0.39	-0.25 (-0.65, 0.15)	0.22	-0.2 (-0.58, 0.17)	0.29	Y
Clostridium beijerinckii	-0.32 (-1.85, 1.2)	0.68	1.94 (-1.75, 5.62)	0.30	-0.15 (-2.09, 1.79)	0.88	-0.26 (-2.09, 1.56)	0.78	Y
Clostridium butyricum	0.36 (-0.26, 0.97)	0.26	-0.82 (-2.31, 0.68)	0.28	0.53 (-0.25, 1.31)	0.18	0.43 (-0.31, 1.17)	0.25	Y
Clostridium sp. L2 50	0.34 (-0.18, 0.86)	0.20	1.04 (-0.21, 2.3)	0.10	0.41 (-0.25, 1.07)	0.23	0.47 (-0.13, 1.08)	0.12	Y
Clostridium symbiosum	-0.12 (-0.39, 0.15)	0.37	0.58 (-0.07, 1.22)	0.08	-0.07 (-0.41, 0.27)	0.69	-0.05 (-0.37, 0.27)	0.76	Y
Anaerofustis stercorihominis	0.08 (-0.27, 0.44)	0.64	-0.72 (-1.58, 0.14)	0.10	0.02 (-0.44, 0.47)	0.95	0.04 (-0.39, 0.47)	0.84	Y
Eubacterium limosum	0.04 (-0.25, 0.32)	0.80	-0.24 (-0.92, 0.44)	0.49	0.13 (-0.23, 0.48)	0.48	0.08 (-0.25, 0.42)	0.63	Y
Anaerostipes caccae	-0.14 (-0.61, 0.33)	0.56	-0.13 (-1.27, 1.02)	0.83	-0.2 (-0.8, 0.4)	0.51	-0.24 (-0.81, 0.33)	0.40	Y

Blautia hydrogenotrophica	-0.06 (-0.52, 0.4)	0.80	-0.29 (-1.4, 0.81)	0.60	-0.09 (-0.67, 0.49)	0.75	-0.1 (-0.65, 0.45)	0.71	Y
Butyrivibrio crossotus	-0.25 (-0.68, 0.17)	0.24	0.67 (-0.36, 1.7)	0.20	-0.41 (-0.95, 0.13)	0.13	-0.4 (-0.91, 0.11)	0.13	Y
Coprococcus eutactus	0.2 (-0.13, 0.54)	0.23	0.38 (-0.44, 1.2)	0.36	0.26 (-0.17, 0.69)	0.24	0.25 (-0.15, 0.65)	0.22	Y
Clostridium difficile	0.17 (-0.22, 0.56)	0.39	0.15 (-0.79, 1.09)	0.75	0.32 (-0.17, 0.81)	0.20	0.26 (-0.2, 0.72)	0.26	Y
Anaerotruncus colihominis	-0.31 (-0.7, 0.07)	0.11	-0.58 (-1.51, 0.35)	0.22	-0.31 (-0.79, 0.18)	0.22	-0.33 (-0.8, 0.13)	0.15	Y
Ruminococcaceae bacterium D16	0.24 (-0.51, 0.99)	0.53	1.46 (-0.35, 3.27)	0.11	0.29 (-0.67, 1.24)	0.55	0.33 (-0.56, 1.22)	0.46	Y
Subdoligranulum variabile	0 (-0.41, 0.41)	1.00	1.18 (0.19, 2.17)	0.02	-0.09 (-0.62, 0.43)	0.72	0.01 (-0.48, 0.5)	0.98	Y
Eubacterium cylindroides	-0.13 (-0.6, 0.34)	0.58	0.45 (-0.69, 1.59)	0.44	-0.26 (-0.85, 0.34)	0.40	-0.11 (-0.67, 0.44)	0.69	Y
Eubacterium dolichum	0.32 (0, 0.64)	0.05	-0.33 (-1.1, 0.45)	0.41	0.43 (0.02, 0.83)	0.04	0.31 (-0.06, 0.68)	0.10	Y
Megasphaera elsdenii	0.43 (-0.59, 1.46)	0.41	1.98 (-0.49, 4.45)	0.12	0.51 (-0.79, 1.81)	0.44	0.52 (-0.71, 1.75)	0.41	Y
Fusobacterium mortiferum	0.18 (-0.08, 0.43)	0.18	0.05 (-0.58, 0.68)	0.87	0.28 (-0.05, 0.61)	0.10	0.28 (-0.03, 0.59)	0.08	Y
Fusobacterium nucleatum	0.02 (-0.33, 0.37)	0.93	0.26 (-0.58, 1.11)	0.54	-0.04 (-0.48, 0.4)	0.86	-0.03 (-0.45, 0.39)	0.89	Y
Fusobacterium ulcerans	0.59 (-0.07, 1.26)	0.08	1.08 (-0.53, 2.7)	0.19	0.8 (-0.05, 1.64)	0.07	0.77 (-0.03, 1.57)	0.06	Y
Fusobacterium varium	0.28 (-0.46, 1.01)	0.46	1.02 (-0.76, 2.8)	0.26	0.5 (-0.44, 1.43)	0.30	0.45 (-0.42, 1.32)	0.31	Y

[1] The 56 microbial SCFA producers were selected from literature and the full list with references is in Table S2. The relative abundance of each SCFAs and the sum of the all three SCFAs were $\log_2$ transformed. The raw counts of each species and the total counts of the 56 species were normalized and $\log_{10}$ transformed [22]. Model was adjusted for age, sex, batch, province, urbanization, income, education, total energy, insoluble fiber, physical activity, smoking, and alcohol intake. β coefficients can be used in the following formula: (2^(β)-1)*100% to estimate the percent change in butyrate associated with per 1 unit increase in $\log_{10}$ of the total relative abundance of SCFAs-producing gut microbiota. None of the false-discovery rate adjusted p-value <0.05.

[2] Microbiota presented in at least 25% of the sample were denoted as non-rare microbiota and kept as continuous variables in linear regression. Microbiota present in less than 25% of the sample were denoted as rare microbiota and included as binary variables (yes/no present in the sample) in linear regression.

Table S10. Sensitivity analysis of the associations between plasma short-chain fatty acids (SCFAs) with body mass index (BMI) and waist-to-height ratio (WHtR)

		**BMI (n=462)**		**WHtR (n=460)**	
	**Mean (SD)**	**β (95% confidence interval)**	**p-value**	**β (95% confidence interval)**	**p-value**
Butyrate/isobutyrate	-0.03 (0.76)	0.36 (-0.02, 0.75)	0.07	0.01 (3E-03, 0.02)	0.01
Valerate	-0.11 (1.74)	0.01 (-0.16, 0.18)	0.91	2E-03 (-2E-03, 4E-03)	0.36
Isovalerate	0.08 (0.94)	0.18 (-0.13, 0.50)	0.24	0.01 (-4E-04, 0.01)	0.07
Total SCFAs	0.06 (0.89)	0.21 (-0.12, 0.54)	0.20	0.01 (2E-04, 0.01)	0.04

Participants who took antibiotics, pre/probiotics, or had diarrhea, irritable bowel syndrome, or inflammatory bowel disease were additionally excluded from the analysis. The mean (SD) for BMI (kg/m^2) and WHtR was 24.01 (3.15) and 0.52 (0.06), respectively. Because the SCFAs abundance were log_2 transformed, the linear model coefficients are interpreted as units of BMI and WHtR associated with a fold increase in SCFAs. Model was adjusted for age, sex, batch, province, urbanization, income, education, total energy, insoluble fiber, physical activity, smoking, and alcohol intake.

Table S11. Sensitivity analysis of the interaction between dietary precursors of short-chain fatty acids (SCFAs) and overweight in models of SCFAs [% change (95% CI)], corresponding to Figure S2[1]

n=462	Butyrate/isobutyrate	Valerate	Isovalerate	Total SCFAs
		**Insoluble fiber (ref. = low)[2]**		
Middle	-14.03 (-27.72, 2.25)	-12.85 (-41.67, 30.19)	-15.74 (-32.15, 4.63)	-15.33 (-31.22, 4.25)
High	0.26 (-16.23, 20)	-13.04 (-42.62, 31.79)	-0.24 (-20.29, 24.85)	0.01 (-19.34, 23.97)
Middle X Overweight	23.97 (-1.94, 56.71)	15.05 (-33.12, 97.89)	28.73 (-3.93, 72.5)	27.46 (-3.41, 68.18)
High X Overweight	1.45 (-19.72, 28.19)	38.81 (-19.22, 138.53)	4.52 (-21.96, 39.98)	4.25 (-21, 37.55)
Interaction p-value[3]	0.133	0.491	0.196	0.185
		**Carbohydrate (ref. = low)[2]**		
Middle	5.21 (-11.46, 25.01)	15.22 (-22.55, 71.4)	5.34 (-15.05, 30.61)	5.7 (-14.14, 29.24)
High	0.26 (-17.8, 22.28)	-11.79 (-44.16, 39.36)	-5.32 (-26.09, 21.28)	-4.07 (-24.21, 20.58)
Middle X Overweight	-1.03 (-21.74, 25.17)	-26.29 (-57.08, 26.59)	-1.48 (-26.49, 32.04)	-1.38 (-25.26, 30.13)
High X Overweight	9.74 (-13.39, 39.05)	17.45 (-31.91, 102.59)	15 (-14.39, 54.5)	14.87 (-13.55, 51.57)
Interaction p-value[3]	0.645	0.238	0.530	0.507
		**Fiber foods (ref. = low)[2]**		
Middle	-4.88 (-19.87, 12.91)	-6 (-36.74, 39.65)	-5.74 (-23.85, 16.68)	-6.05 (-23.16, 14.87)
High	-3.21 (-18.95, 15.58)	20.39 (-20.08, 81.36)	-0.83 (-20.48, 23.68)	-1.38 (-19.89, 21.42)
Middle X Overweight	-9.08 (-27.98, 14.79)	-6.31 (-45.3, 60.49)	-15.5 (-36.78, 12.94)	-14.14 (-34.48, 13.29)
High X Overweight	-5.21 (-25.03, 19.83)	-22.43 (-54.86, 33.31)	-6.27 (-30, 25.49)	-6.05 (-28.8, 23.97)
Interaction p-value[3]	0.724	0.635	0.517	0.660

[1] Participants who took antibiotics, pre/probiotics, or had diarrhea, irritable bowel syndrome, or inflammatory bowel disease were additionally excluded from the analysis. BMI ≥24 kg/m^2. The abundance of each SCFAs and the sum of the all three SCFAs were $\log_2$ transformed. The % change relative to the reference level was calculated using the following formula: $(2^{(\beta)}-1)*100\%$, where β was the linear model coefficient. Model was adjusted for age, sex, batch, province, urbanization, income, education, total energy, insoluble fiber, physical activity, smoking, and alcohol intake. *, % change p-value<0.05, **, % change p-value<0.01.

[2] Dietary intakes were measured by 3-consecutive 24h dietary recalls and household food inventories. High-fiber foods was calculated as the sum of whole grains, legumes, starchy roots, vegetables, mushrooms/seaweeds, fruits, nuts/seeds. Insoluble fiber, carbohydrate, and high-fiber food score were categorized by tertiles to represent low, middle, and high intakes.

[3] The statistical significance of the interaction term between dietary factors and overweight was estimated using a Wald test that compared models with and without the interaction term.

Table S12. Sensitivity analysis of the interaction between dietary precursors of short-chain fatty acids (SCFAs) and abdominal obesity in models of SCFAs [% change (95% CI)], corresponding to Figure S3[1]

n=460	Butyrate/isobutyrate	Valerate	Isovalerate	Total SCFAs
**Insoluble fiber (ref. = low)[2]**				
Middle	-18.6 (-33.56, -0.27)	-16.01 (-47.74, 34.99)	-19.59 (-37.68, 3.76)	-19.34 (-36.71, 2.81)
High	0.7 (-18.25, 24.06)	-13.12 (-46.63, 41.44)	1.15 (-22.15, 31.42)	1.4 (-21, 29.24)
Middle X Abdominal obesity	26.33 (-0.89, 61.04)	16.67 (-33.83, 105.71)	28.24 (-5.44, 73.92)	27.46 (-4.07, 70.53)
High X Abdominal obesity	0.32 (-21.43, 28.09)	22.74 (-30.65, 117.24)	1.3 (-25.45, 37.67)	1.4 (-24.21, 35.66)
Interaction p-value[3]	0.099	0.762	0.201	0.132
**Carbohydrate (ref. = low)[2]**				
Middle	2.77 (-16.34, 26.24)	-19.57 (-50.03, 29.46)	0.54 (-22.3, 30.11)	0.7 (-21, 28.34)
High	13.75 (-9.6, 43.14)	-42.92 (-66.46, -2.87)*	12.45 (-15.68, 49.97)	11.73 (-14.74, 47.43)
Middle X Abdominal obesity	3.5 (-18.9, 32.08)	36.39 (-22.42, 139.77)	6.67 (-21.41, 44.79)	6.44 (-19.89, 42.41)
High X Abdominal obesity	-10.79 (-30.57, 14.63)	110.58 (17.9, 276.14)*	-13.63 (-36.92, 18.25)	-11.73 (-34.48, 18.92)
Interaction p-value[3]	0.473	0.042	0.396	0.530
**High-fiber foods (ref. = low)[2]**				
Middle	-13.95 (-29.69, 5.31)	-1.38 (-38.39, 57.87)	-18.65 (-36.82, 4.75)	-17.64 (-35.38, 4.25)
High	-5.29 (-23.05, 16.58)	7.9 (-33.49, 75.06)	-2.38 (-24.73, 26.6)	-2.73 (-24.21, 23.97)
Middle X Abdominal obesity	7.96 (-15.22, 37.47)	-10.78 (-49.19, 56.66)	9.64 (-18.98, 48.36)	9.43 (-17.64, 45.4)
High X Abdominal obesity	-2.45 (-23.42, 24.28)	1.1 (-42.48, 77.7)	-4.53 (-29.49, 29.26)	-4.07 (-27.8, 27.46)
Interaction p-value[3]	0.701	0.893	0.669	0.840

[1] Participants who took antibiotics, pre/probiotics, or had diarrhea, irritable bowel syndrome, or inflammatory bowel disease were additionally excluded from the analysis. Waist-to-height ratio ≥0.5. The abundance of each SCFAs and the sum of the all three SCFAs were $\log_2$ transformed. The % change relative to the reference level was calculated using the following formula: $(2^{(\beta)}-1)*100\%$, where β was the linear model coefficient. Model was adjusted for age, sex, batch, province, urbanization, income, education, total energy, insoluble fiber, physical activity, smoking, and alcohol intake. *, % change p-value<0.05, **, % change p-value<0.01.

[2] Dietary intakes were measured by 3-consecutive 24h dietary recalls and household food inventories. High-fiber foods was calculated as the sum of whole grains, legumes, starchy roots, vegetables, mushrooms/seaweeds, fruits, nuts/seeds. Insoluble fiber, carbohydrate, and high-fiber food score were categorized by tertiles to represent low, middle, and high intakes.

[3] The statistical significance of the interaction term between dietary factors and abdominal obesity was estimated using a Wald test that compared models with and without the interaction term.

Table S13. Sensitivity analysis of the association between the overall and the total relative abundance of 56 microbial short-chain fatty acid (SCFA) producers with body mass index (BMI) and waist-to-height-ratio (WHtR)

			Overall[1]		Total[2]	
	**n**	**Mean (SD)**	**R^2**	**P-value**	**β (95% CI)**	**P-value**
BMI	192	24.38 (3.12)	0.008	0.13	-0.95 (-2.82, 0.93)	0.32
WHtR	191	0.53 (0.06)	0.007	0.20	-0.03 (-0.06, 0.01)	0.14

Participants who took antibiotics, pre/probiotics, or had diarrhea, irritable bowel syndrome, or inflammatory bowel disease were additionally excluded from the analysis. The raw counts of each species and the total counts of the 56 species were normalized and $\log_{10}$ transformed [22]. Model was adjusted for age, sex, batch, province, urbanization, income, education, total energy, insoluble fiber, physical activity, smoking, and alcohol intake.

[1] R^2 and p-value were calculated using permutational multivariate analysis of variance (PERMANOVA) of all 56 species.

[2] Linear regression was performed on the total relative abundance of the 56 species.

References

1. Barcenilla, A.; Pryde, S.E.; Martin, J.C.; Duncan, S.H.; Stewart, C.S.; Henderson, C.; Flint, H.J. Phylogenetic relationships of butyrate-producing bacteria from the human gut. *Appl. Environ. Microbiol.* **2000**, *66*, 1654-1661.
2. LeBlanc, J.G.; Chain, F.; Martín, R.; Bermúdez-Humarán, L.G.; Courau, S.; Langella, P. Beneficial effects on host energy metabolism of short-chain fatty acids and vitamins produced by commensal and probiotic bacteria. *Microbial cell factories* **2017**, *16*, 79.
3. Louis, P.; Flint, H.J. Formation of propionate and butyrate by the human colonic microbiota. *Environmental microbiology* **2017**, *19*, 29-41.
4. Reichardt, N.; Duncan, S.H.; Young, P.; Belenguer, A.; Leitch, C.M.; Scott, K.P.; Flint, H.J.; Louis, P. Phylogenetic distribution of three pathways for propionate production within the human gut microbiota. *The ISME journal* **2014**, *8*, 1323.
5. Vital, M.; Howe, A.C.; Tiedje, J.M. Revealing the bacterial butyrate synthesis pathways by analyzing (meta) genomic data. *MBio* **2014**, *5*, e00889-00814.
6. Pryde, S.E.; Duncan, S.H.; Hold, G.L.; Stewart, C.S.; Flint, H.J. The microbiology of butyrate formation in the human colon. *FEMS microbiology letters* **2002**, *217*, 133-139.
7. Louis, P.; Young, P.; Holtrop, G.; Flint, H.J. Diversity of human colonic butyrate-producing bacteria revealed by analysis of the butyryl-CoA: acetate CoA-transferase gene. *Environmental microbiology* **2010**, *12*, 304-314.
8. Muñoz-Tamayo, R.; Laroche, B.; Walter, É.; Doré, J.; Duncan, S.H.; Flint, H.J.; Leclerc, M. Kinetic modelling of lactate utilization and butyrate production by key human colonic bacterial species. *FEMS microbiology ecology* **2011**, *76*, 615-624.
9. Duncan, S.H.; Louis, P.; Flint, H.J. Lactate-utilizing bacteria, isolated from human feces, that produce butyrate as a major fermentation product. *Appl. Environ. Microbiol.* **2004**, *70*, 5810-5817.
10. Belenguer, A.; Duncan, S.H.; Calder, A.G.; Holtrop, G.; Louis, P.; Lobley, G.E.; Flint, H.J. Two routes of metabolic cross-feeding between Bifidobacterium adolescentis and butyrate-producing anaerobes from the human gut. *Appl. Environ. Microbiol.* **2006**, *72*, 3593-3599.
11. Patterson, A.M.; Mulder, I.E.; Travis, A.J.; Lan, A.; Cerf-Bensussan, N.; Gaboriau-Routhiau, V.; Garden, K.; Logan, E.; Delday, M.I.; Coutts, A.G. Human gut symbiont Roseburia hominis promotes and regulates innate immunity. *Frontiers in immunology* **2017**, *8*, 1166.
12. Duncan, S.H.; Holtrop, G.; Lobley, G.E.; Calder, A.G.; Stewart, C.S.; Flint, H.J. Contribution of acetate to butyrate formation by human faecal bacteria. *British Journal of Nutrition* **2004**, *91*, 915-923.
13. Crost, E.H.; Tailford, L.E.; Le Gall, G.; Fons, M.; Henrissat, B.; Juge, N. Utilisation of mucin glycans by the human gut symbiont Ruminococcus gnavus is strain-dependent. *PloS one* **2013**, *8*, e76341.
14. Wrzosek, L.; Miquel, S.; Noordine, M.-L.; Bouet, S.; Chevalier-Curt, M.J.; Robert, V.; Philippe, C.; Bridonneau, C.; Cherbuy, C.; Robbe-Masselot, C. Bacteroides thetaiotaomicron and Faecalibacterium prausnitzii influence the production of mucus glycans and the development of goblet cells in the colonic epithelium of a gnotobiotic model rodent. *BMC biology* **2013**, *11*, 61.
15. Laverde Gomez, J.A.; Mukhopadhya, I.; Duncan, S.H.; Louis, P.; Shaw, S.; Collie-Duguid, E.; Crost, E.; Juge, N.; Flint, H.J. Formate cross-feeding and cooperative metabolic interactions revealed by transcriptomics in co-cultures of acetogenic and amylolytic human colonic bacteria. *Environmental microbiology* **2019**, *21*, 259-271.

16. Sakon, H.; Nagai, F.; Morotomi, M.; Tanaka, R. Sutterella parvirubra sp. nov. and Megamonas funiformis sp. nov., isolated from human faeces. *International journal of systematic and evolutionary microbiology* **2008**, *58*, 970-975.
17. Kelly, W.J.; Henderson, G.; Pacheco, D.M.; Li, D.; Reilly, K.; Naylor, G.E.; Janssen, P.H.; Attwood, G.T.; Altermann, E.; Leahy, S.C. The complete genome sequence of Eubacterium limosum SA11, a metabolically versatile rumen acetogen. *Standards in genomic sciences* **2016**, *11*, 26.
18. Durand, M.; Bernalier, A.; Cummings, J.; Rombeau, J.; Sakata, T. Reductive acetogenesis in animal and human gut. *Physiological and clinical aspects of shortchain fatty acids* **1993**, 107-117.
19. Sato, T.; Matsumoto, K.; Okumura, T.; Yokoi, W.; Naito, E.; Yoshida, Y.; Nomoto, K.; Ito, M.; Sawada, H. Isolation of lactate-utilizing butyrate-producing bacteria from human feces and in vivo administration of Anaerostipes caccae strain L2 and galacto-oligosaccharides in a rat model. *FEMS microbiology ecology* **2008**, *66*, 528-536.
20. Sivieri, K.; Morales, M.L.V.; Adorno, M.A.T.; Sakamoto, I.K.; Saad, S.M.I.; Rossi, E.A. Lactobacillus acidophilus CRL 1014 improved “gut health” in the SHIME® reactor. *BMC gastroenterology* **2013**, *13*, 100.
21. Derrien, M.; Vaughan, E.E.; Plugge, C.M.; de Vos, W.M. Akkermansia muciniphila gen. nov., sp. nov., a human intestinal mucin-degrading bacterium. *International journal of systematic and evolutionary microbiology* **2004**, *54*, 1469-1476.
22. Jones, R.B.; Zhu, X.; Moan, E.; Murff, H.J.; Ness, R.M.; Seidner, D.L.; Sun, S.; Yu, C.; Dai, Q.; Fodor, A.A. Inter-niche and inter-individual variation in gut microbial community assessment using stool, rectal swab, and mucosal samples. *Scientific reports* **2018**, *8*, 1-12.
